# Rotational Dynamics of a Protein under Shear Flow Studied by the Eckart Frame Formalism

**DOI:** 10.1021/acs.jpcb.3c02324

**Published:** 2023-08-09

**Authors:** Petra Papež, Franci Merzel, Matej Praprotnik

**Affiliations:** † Theory Department, National Institute of Chemistry, Hajdrihova 19, SI-1001 Ljubljana, Slovenia; ‡ Department of Physics, Faculty of Mathematics and Physics, University of Ljubljana, Jadranska 19, SI-1000 Ljubljana, Slovenia

## Abstract

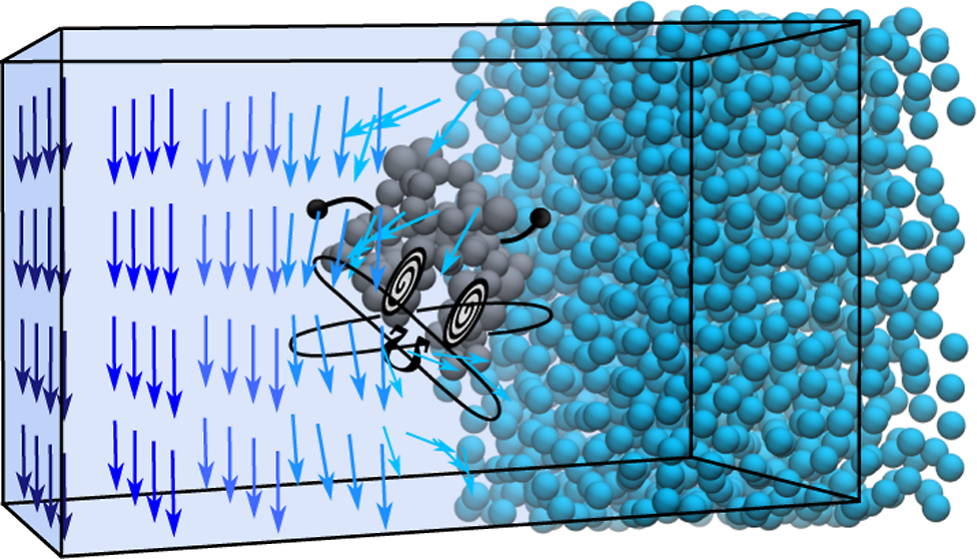

Proteins are natural polymers that play an essential role in both living organisms and biotechnological applications. During certain bioprocessing steps, they can be exposed to significant mechanical stress induced by, for example, shear flow or sonication, resulting in reduced therapeutic efficacy, aggregation, or even a loss of activity. For this reason, there is a need to understand and determine the susceptibility of the protein activity to the experienced mechanical stress. To acquire this knowledge, it is necessary to study the rotational dynamics of the protein. Commonly, the rotational dynamics of soft molecules is interpreted based on a theoretical analysis performed in an inertial laboratory frame. However, the obtained angular velocity mixes pure rotations and vibrations with angular momentum, consequently lacking a clear dynamical interpretation. On the other hand, the use of the noninertial internal Eckart frame allows the determination of pure angular velocity as it minimizes the coupling between the rotational and vibrational degrees of freedom. In the present work, by conducting open-boundary molecular dynamics simulations and exploiting the Eckart frame formalism, we study the rotational dynamics of a small protein under the shear flow of various strengths. Our results show that the angular velocity increases nonlinearly with increasing shear rate. Furthermore, the protein gains vibrational angular momentum at higher shear rates, which is reflected in the higher angular velocity computed by employing the Eckart frame formalism and confirmed by analysis of the contributions to the total kinetic energy of the biomolecule.

## 1 Introduction

Proteins are biopolymers consisting of chains of amino acids. The amino acid sequence defines the unique 3D structure of the protein and is related to its function. Proteins in their native (folded) state are an indispensable part of biological processes in living organisms. For instance, these macromolecules are structural components of cells and tissues; they act as chemical messengers and catalysts for biochemical reactions, support regulation and expression of DNA and RNA and immune function,^1^ and transport cargo around the body as biological machines that convert chemical energy into mechanical work.^2^ In addition, these biopolymers are pivotal not only in biological processes but also in biotechnological applications.^1,3^ Changes in their native structure can result in the loss of biological function or catalytic activity, reduced therapeutic efficacy, and formation of insoluble aggregates (commonly associated with often fatal human diseases, which include Parkinson’s and Alzheimer’s diseases).^1,4^

Several factors affecting protein stability and causing conformational changes have been extensively studied and are now well understood. These factors include temperature, pressure, pH, ion concentration, or the presence of molecular agents,^5^ to name just a few. However, less focus was on protein conformational changes caused by mechanical stress, such as hydrodynamic shear. Understanding the influence of shear on the stability (and consequently activity) of proteins is of great importance in bioprocessing as during specific steps, such as centrifugation, fractionation, pumping, and ultrafiltration, solutions of these biomolecules are subjected to shear stresses that can cause loss of function and aggregation.^1,3^ Intriguingly, dissimilar results are reported in the literature, considering the effect of shear on protein stability and function. For example, Charm and Wong^6^ reported that catalase and carboxypeptidase lose their activity when pumped through a narrow cylindrical viscometer due to the shear-induced breakage of the tertiary structure. Additionally, Charm and Wong^6^ proposed an equation to compute the expected shear inactivation of the protein solution flowing through a capillary tube. On the contrary, Thomas and Dunnill^7^ did not find significant losses in catalase activity in the presence of urea and also in capillary rheometers at shear rates up to 10^6^ s^–1^. Jaspe and Hagen^8^ showed that there is no proof that even shear rates up to ∼2 × 10^5^ s^–1^ destabilize cytochrome *c*. Based on the developed elementary model, Jaspe and Hagen^8^ estimated that the shear rate in water must be extraordinarily high (∼10^7^ s^–1^) to observe the denaturation of small globular proteins (about 100 amino acids). Similarly, Duerkop et al.^9^ hypothesized that shear rates of up to 10^8^ s^–1^ should not be considered harmful to an average-sized protein involved in bioprocesses. Nevertheless, in an in situ study of the shear effect on aqueous insulin, Bekard and Dunstan^10^ showed that the deformation of insulin is shear-dependent. Furthermore, by performing in situ measurements, Ashton et al.^11^ identified reversible, shear-induced conformational changes of lysozyme in water and glycerol solutions.

Many studies performed to evaluate the effects of shearing on protein structure and activity^8,11–15^ show that proteins do not exhibit universal behavior in shear flow. Nevertheless, they still lack a clear dynamic interpretation concerning the energy dissipation into the internal degrees of freedom that affect the conformational changes of the protein. To address this question from a dynamical perspective, it would be beneficial to thoroughly study the rotational and vibrational behaviors of the biomolecule when subjected to shear flow. However, as reported by Sablić et al.,^16^ the rotational dynamics of soft molecules obtained by the standard analysis performed in the inertial laboratory frame is misinterpreted. Apart from the rotational contribution, the obtained angular velocity also includes the vibrational one, leading to an unclear dynamical explanation. Separating the system’s rotations from its vibrations can be achieved by employing Eckart frame formalism. This formalism is commonly applied to describe the infrared and Raman spectra of small molecules,^17,18^ and recently, Sablić et al.,^16^ Jaramillo-Cano et al.,^19^ and Toneian et al.^20^ showed that the Eckart corotating frame is robust enough to allow investigation of the complex dynamics of the macromolecules under shear flow.

Following up on the work conducted by Sablić et al.^16^ on generic star polymers, the first aim of this study is to apply the Eckart frame formalism to investigate the dynamics of the biologically relevant molecule in water under shear flow. As the benchmark protein, we choose ubiquitin and perform the open-boundary molecular dynamics (OBMD) simulations and use the coarse-grained (CG) Martini 3 model^21^ combined with an elastic network (EN). Using the OBMD simulation approach allows us to impose the external boundary condition (i.e., shear flow) without changing Newton’s equations of motion, and the CG model is sufficient to address the generic physical properties of the protein. In addition, the OBMD is also more flexible in defining external boundary conditions compared to the nonequilibrium molecular dynamics simulations.^22^ Using the laboratory and Eckart frames, we compute and compare the angular velocity of the protein subjected to a shear flow of various strengths. Employing the Eckart frame formalism, we correctly determine the contributions of different types of motion (i.e., translational, rotational, and vibrational, with and without angular momentum) to the total kinetic energy of the biomolecule. As noted by Sablić et al.,^16^ this analysis can be complementary to the normal-mode analysis of the vibrations within the framework of the theory of molecular vibrations.^23^ For this reason, based on the unveiled rotational and vibrational motions, the Eckart frame formalism could also pave the way for use in protein activity interference.

A two-dimensional flow may be defined as a linear superposition of varying amounts of rotational and elongational flows. Generally, the amount of polymer deformation strongly depends on its nature.^24–31^ However, in a purely rotational flow, only rotation (without induced deformation) is expected, whereas in a purely elongational flow, large deformations are expected.^25,32–36^ On the other hand, in the simple shear flow, the magnitudes of the rotational and elongational components are equal.^25^ As argued by de Gennes,^25^ in the simple shear flow, the polymers do not attain a stable, strongly stretched state but rather undergo a tumbling motion with large fluctuations in their extension.^33,37,38^ Similarly, Alexander-Katz et al.^39^ observed the unfolding/refolding cycles of the polymeric globule. Therefore, the second aim of this paper is to observe the characteristic dynamics of exchanging stretched and coiled states. To achieve this, we compute the time evolution of the radius of gyration^40^ during the production run of the simulation and visualize configurations that best represent the conformational dynamics of the biomolecule. Finally, we also calculate angular velocity using the laboratory frame (ω_3_), the Eckart frame formalism (Ω_3_), and contributions to the total kinetic energy and compare them with those determined when the EN is unaltered.

## 2 Theoretical Background and Methodology

**Figure 1:**
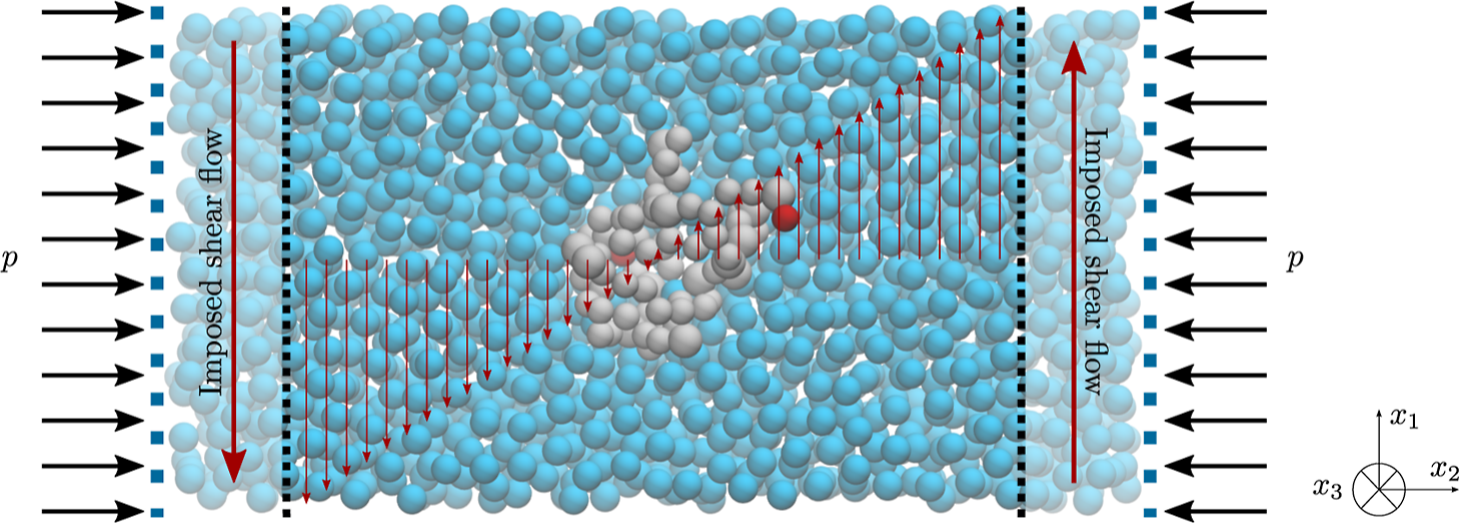
Schematic representation of the simulated system showing the protein ubiquitin immersed in water under shear flow.

Using molecular dynamics simulations, we study the effect of shear on the rotational and conformational dynamics of ubiquitin. The protein (modeled as the Martini 3 model^21^) is placed at the center of the simulation box, surrounded by CG water molecules and subjected to the shear flow of various strengths. A schematic representation of the simulated system is depicted in Figure 1. For better visibility, the protein is represented by CG backbone particles and colored gray, while the surrounding medium is colored blue. The coordinate vectors *x*_1_, *x*_2_, and *x*_3_ in Figure 1 denote the flow, gradient, and vorticity directions, respectively.

To inspect the rotational and conformational dynamics of the protein immersed in water under shear flow, the OBMD method is used, which imposes external boundary conditions of a constant normal load and shear flow onto the system without altering Newton’s equations of motion. As an illustration, in Figure 1, the imposition of the first boundary condition is represented by black arrows on the sides of the simulation box, while the imposition of the second boundary condition is depicted by the large red arrow in the side (buffer) regions of the simulation domain. The Martini protein model and the OBMD method used are discussed in the following subsections.

### 2.1 CG Model of Ubiquitin

**Figure 2:**
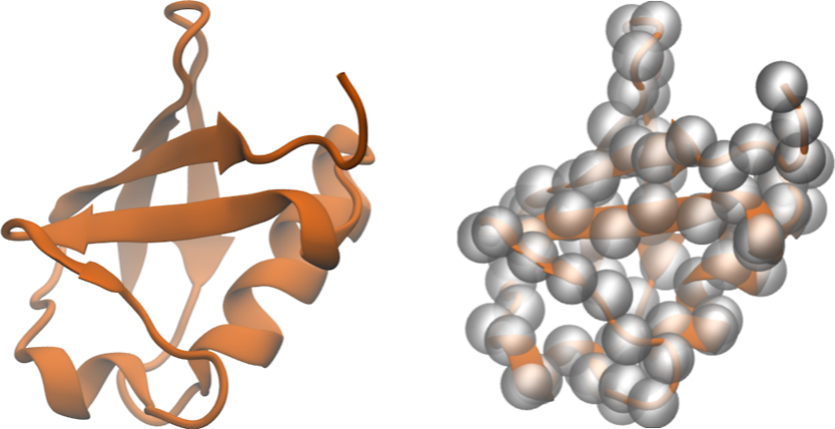
CG Martini model of ubiquitin. The figure on the left is a cartoon representation of the protein’s atomistic structure, while the right figure depicts the backbone beads of the constructed CG protein model, describing the underlying atomistic structure.

To obtain the CG Martini 3 model of ubiquitin, the initial atomistic structure (PDB entry 1UBQ(41)) of the protein is converted to the CG Martini 3 model using Martinize2 available at https://github.com/marrink-lab/vermouth-martinize. The initial atomistic structure and its CG representation are shown in Figure 2. To maintain the secondary and tertiary structures, an EN of additional harmonic bonds is typically applied to the CG backbone particles of the protein with an elastic bond constant of 550 kJ mol^–1^ nm^–2^, where the lower and upper elastic bond cutoffs are set to 0.5 and 0.9 nm, respectively.

As large-scale structural changes (such as unfolding) are disfavored, when the standard EN model is used, we improve the method of constraining by defining the thresholds for the distances between the CG bead pairs that form EN and are connected by harmonic bonds. When the predefined threshold values are exceeded, the harmonic bonds of EN irreversibly break, and the CG bead pairs are added to the Verlet list
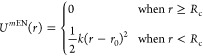
1where *k* and *r*_0_ are the force constant and equilibrium distance, respectively, defined in the Martini v3.0.0 force field, while *R*_c_ stands for a predefined cutoff value. The latter is determined based on the monitoring of the protein structure compactness (i.e., the radius of gyration^40^) during the equilibrium simulation (i.e., zero shear condition) of length 50 ns, where the same adjustment of EN is implemented. Therefore, based on the observation of protein structure conservation in equilibrium (i.e., zero shear), the *R*_c_ is set to 1.35 times the distance between the CG backbone particles of EN defined in the Martini v3.0.0 force field. In addition, to prevent the protein from diffusing through the open ends of the simulation box or over the edges, where the periodic boundary conditions are implemented, an additional spring is added to hold the protein center of mass close to the center of the simulation box.

### 2.2 Open-Boundary Molecular Dynamics

The shear flow of various strengths is imposed by using OBMD. OBMD is a simulation technique that opens the boundaries of the simulated system and allows the exchange of momentum, energy, and mass between the system and its surroundings.^22,42–44^ Accordingly, the simulation box is opened in one direction, while the periodic boundary conditions are imposed on the remaining ones. The simulation box is divided into three regions, with the central region [i.e., the region of interest (ROI)] enclosed by two buffer regions. Buffers serve as particle reservoirs, from which particles are deleted and inserted into the system. The number of particles in the buffers is controlled by the feedback algorithm given by Δ*N*_B_ = (δ*t*/τ_B_) (⟨*N*_B_⟩ – *N*_B_). Here, τ_B_ represents the characteristic relaxation time of the buffers [usually of the order τ_B_ ∼ *O*(100δ*t*)], δ*t* stands for the time step, while ⟨*N*_B_⟩ and *N*_B_ denote the desired number of particles inside the buffer and the current number of particles inside the buffer, respectively. When Δ*N*_B_ < 0, the particles need to be deleted from the system. To do this, the particles are first left to diffuse over the outer boundary of the buffer and then erased. On the contrary, when Δ*N*_B_ > 0, new particles need to be inserted into the buffer region. The insertion of new particles is carried out by the iterative algorithm called USHER.^45,46^ Additionally, another important feature of buffers is the imposition of the external boundary conditions onto the ROI. To this end, the OBMD uses an additional external force **F**^ext^ in the buffer domains. In pursuance of expressing this force, we must first define the linear momentum conservation law: ∂(ρ**v**)/δ*t* = −∇·**J**^*P*^ and express the linear momentum flux tensor
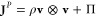
2Above, ρ and **v** represent the density and velocity, respectively. In eq 2, Π is the mean contribution to the pressure tensor. The pressure tensor is commonly defined as Π = (*p* + π)**I** + Π^*S*^, where *p* stands for the pressure of the system, **I** is the identity matrix, π represents the isotropic stress (π = −ζ∇·**v**), and Π^*S*^ is the traceless symmetric tensor, expressed as Π_αβ_^*S*^ = −η(∂_α_*v*_β_ + ∂_β_*v*_α_ – 2∂_γ_*v*_γ_δ_αβ_/*D*). ζ and η are bulk and dynamic viscosity, respectively, while *D* denotes the spatial dimension.^47,48^ Afterward, **F**^ext^ is computed from the momentum balance for the surface *A*, i.e., for the area of the interface buffer-ROI
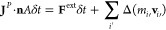
3In eq 3, **F**^ext^ = ∑_*i*∈*B*_**f**_*i*_^ext^, where *i* runs over all the particles that are within buffer regions, while *i*′ runs over all particles that have been inserted or deleted from the system in the last time step δ*t*. Thus, the momentum change is expressed as Δ(*m*_*i*′_**v**_*i*′_) = ±*m*_*i*′_**v**_*i*′_ if particle *i*′ is inserted (+) or deleted (−). The unit vector normal to the interface buffer-ROI (pointing toward the center of the ROI) is denoted by **n**, while **J**^*P*^ stands for the already defined momentum flux tensor (eq 2). If one aims to simulate a system under a constant normal load, then the momentum flux tensor given by eq 2 simplifies to *J*_*ij*_^*P*^ = *p*δ_*ij*_. However, in this work, not only the constant normal load but also the shear flow is imposed on the open system (see Figure 1). Therefore, the components of the corresponding momentum flux tensor are *J*_11_^*P*^ = ργ̇^2^*x*_2_^2^ + *p*, *J*_12_^*P*^ = *J*_21_^*P*^ = −ηγ̇, *J*_22_^*P*^ = *J*_33_^*P*^ = *p*, and *J*_13_^*P*^ = *J*_31_^*P*^ = *J*_23_^*P*^ = *J*_32_^*P*^ = 0, where γ̇ represents the shear rate of the flow and *x*_2_ denotes the coordinate in the open direction of the system (i.e., gradient direction). Accordingly, the momentum flux part of the external force is expressed as **J**^*P*^·**n** = *J*_22_^*P*^·**n** + *J*_12_^*P*^·**t**, where the unit vector **t** points in the direction of the shear flow (i.e., in the *x*_1_-direction), and it is perpendicular to the already introduced unit vector **n**. In our simulations, where shear flow and constant normal load are imposed, the external force can be decomposed into normal and tangential contributions and expressed as **F**^ext^ = *F*_∥_^ext^**n** + *F*_⊥_^ext^**t**. Hence, the force on the particle within the buffer is
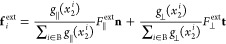
4In eq 4, *g*_∥_ and *g*_⊥_ are weighting functions that distribute the contributions to the external force in normal (i.e., **n**) and tangential (i.e., **t**) directions, respectively.^44^

As OBMD involves the transfer of momentum, the indispensable part is also the linear momentum conserving thermostat.

### 2.3 Dissipative Particle Dynamics Thermostat

This requirement is met by the dissipative particle dynamics (DPD) thermostat because its equations conserve linear momentum and correctly reproduce the hydrodynamic behavior.^49–51^ In this work, the conservative force **F**_*i*_ acting on the *i*th particle of the simulated system is obtained as the negative gradient of the potential energy defined by the applied force field. Additionally, the chosen *r*-dependent weight functions ω^*D*^(*r*) and ω^*R*^(*r*) that satisfy the fluctuation–dissipation theorem are defined as
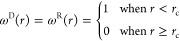
5where *r*_c_ and *r* stand for the cutoff radius and interparticle distance, respectively. ω^D^(*r*) and ω^R^(*r*) are part of the dissipative and random forces, respectively, which together form the DPD thermostat.

Due to the expected heating of the system during its exposure to high shear rates, we modify the standard DPD thermostat by controlling its random contribution to the force as suggested by Sablić et al.^44^

Conducting the OBMD simulations and employing the adaptive DPD thermostat permit us to investigate the dynamics and eventual structural evolution of a biomolecule subjected to shear flow.

### 2.4 Inertial Laboratory Frame

We start our analysis of rotational dynamics by decomposing the total kinetic energy into translational, rotational, and vibrational. According to the standard approach, which is based on the inertial frame, the (apparent) angular velocity is computed as
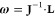
6where **J** and **L** represent the moment of inertia tensor and the angular momentum of the rotating molecule, respectively. The former is given by
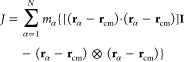
7and the latter is expressed as
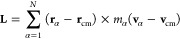
8where **r**_α_ and **v**_α_ are the position and velocity, respectively, of an α particle with mass *m*_α_ that builds up the protein molecule, while **r**_cm_ and **v**_cm_ are the position and velocity of biomolecule’s center of mass, respectively. In eq 7, **I** stands for the 3 × 3 identity matrix. However, eq 6 is only valid for the rigid-body rotation, while in this study, we are dealing with the nonrigid molecules. Therefore, the time evolution of the position of the protein’s α particle involving rigid translation, rotation, and vibrational type of motion is given by
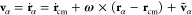
9Above, **ṽ**_α_ denotes the vibrational motion that is angular momentum free.

The kinetic energy of the rotating and vibrating molecule is correspondingly
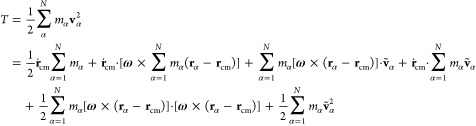
10From
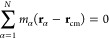
11and
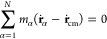
12where (**ṙ**_α_ – **ṙ**_cm_) is expressed from eq 9, it follows
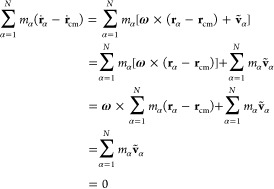
13By inspecting terms on the rhs of eq 10 and using eqs 11 and 13, it is clear that the second and fourth terms are zero, and the third term
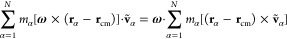
14represents the Coriolis coupling. Since all the rotational contribution is already collected in the apparent angular velocity **ω**, the Coriolis coupling term is zero, which is consistent with the fact that in the laboratory frame, no noninertial forces are present. Hence, the Coriolis coupling term is zero in the laboratory frame. The total kinetic energy is finally expressed as
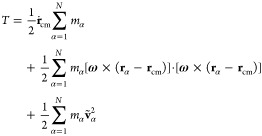
15where terms on the rhs of the eq 15 are decomposed into translational (*T*_trans_), rotational (*T*_rot_^lab^), and vibrational (*T*_vib_^lab^), respectively.

Following the above analysis in the inertial laboratory frame, it is not possible to distinguish between pure rotations and vibrations with angular momentum since they are hidden together in the apparent angular velocity **ω**. Contrary to the case of the inertial laboratory frame, the Coriolis coupling is nonzero in a noninertial coordinate system, defining a noninertial force. Exploiting the noninertial internal Eckart frame allows this coupling to be minimized.^52^ Any other noninertial coordinate system will give a larger Coriolis coupling.

### 2.5 Noninertial Internal Eckart Frame

The Eckart frame is a noninertial frame that corotates with the molecule.^17,52^ It permits the unveiling of vibrations with and without angular momentum, thus allowing the determination of pure angular velocity **Ω**.

In the noninertial Eckart frame, the total kinetic energy of the biomolecule is expressed as^16,53–56^
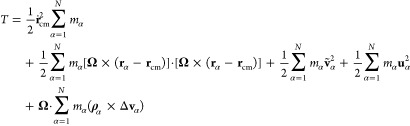
16where the Eckart angular velocity **Ω** is defined as
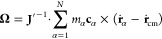
17and **J**′ is given by
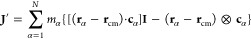
18

In the above equations, **c**_α_ stands for the equilibrium positions of the beads in the instantaneous noninertial Eckrat frame, **ρ**_α_ denotes the displacement vector describing instantaneous positions of particle α relative to the reference position, while the sum of **ṽ**_α_ and **u**_α_, standing for the angular motion free part (the same as in eq 9) and angular motion part of the vibrational contribution, respectively, defines Δ**v**_α_. Therefore, terms on the rhs of eq 16 correspond to the translational (*T*_trans_) and rotational (*T*_rot_^Eck^) contributions to the total kinetic energy, followed by two vibrational contributions. The first one arises from the angular free part of vibrational motion (*T*_vib-non-ang_^Eck^), while the second describes the angular part of vibrations (*T*_vib-ang_^Eck^). The last term on the rhs of eq 16 is the Coriolis coupling (*T*_Cori_^Eck^).

Finally, a comparison of the kinetic energy expressions obtained in the laboratory and Eckart frames yields the following relations
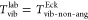
19and
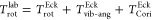
20where the translational contribution to the total kinetic energy is the same in both frames.^16,18^

## 3 Computational Details

In order to properly address the rotational dynamics of the protein under shear flow, we chose a relatively small protein ubiquitin, modeled as described in Section 2.1, and performed the OBMD simulation in water using the Eckart frame formalism.

In accordance with the OBMD, the simulation box is divided into three regions, i.e., two buffer regions surrounding the ROI. At the center of the simulation box (or at the center of the ROI) is the protein molecule immersed in water (Figure 1). Information on the secondary structure classification of the protein backbone from the structure is provided from the DSSP database available at https://github.com/cmbi/dssp/releases/tag/2.3.0. The geometry of the protein molecule is constrained using RATTLE.^57^ Equations of motion are integrated using the velocity Verlet algorithm^58^ with an integration time step δ*t* = 0.02 ps at a temperature of 300 K. All simulations are performed using the Martini v3.0.0 force field and the ESPResSo++ simulation package.^59^ To describe the nonbonded interactions, the Lennard-Jones 12-6 potential energy function with a cutoff value of 1.1 nm is used. Apart from the Lennard-Jones interactions, long-range interactions of charged groups are treated with the Coulombic energy function with a relative permeability of ε_r_ = 15 and a cutoff distance of 1.1 nm. In the pursuit of the first objective of this study (i.e., to inspect the effect of the shear flow on the rotational dynamics of the biomolecule), the Martini protein combined with the EN is immersed in the rectangular box of Martini water. The dimensions of the box are set to 12.6 × 6.2 × 6.2 nm^3^, and several simulations of 20 ns length are performed, of which the last 19 ns is used for the production run. In order to observe the dynamics of the exchanging stretched and coiled states, some simulations are prolonged, and the dimension of the simulation box coinciding with the direction of the imposed shear flow is increased as large fluctuations in the extension of the protein are expected in this direction. Therefore, simulations of 100 ns length are performed, where the last 75 ns is used for the production run, and the dimensions of the simulation box are set to 10 × 15 × 10 nm^3^.

## 4 Results and Discussion

The rotation of a sphere immersed in a shear flow field was studied by Einstein.^60^ As he established, the angular velocity of a spherical particle is constant and given by ω = γ̇/2, where γ̇ stands for the shear rate, assuming the no-slip boundary condition, absence of fluid and particle inertia, and gravity and Brownian motion. This implies that the angular velocity of the sphere is independent of its size and the viscosity of a fluid in which it is immersed.^60,61^ On the other hand, axisymmetric ellipsoids and spheroids in shear flow exhibit a more complex nature. The rotation of an axisymmetric inertia-free spheroidal particle in the simple shear flow was described by Jeffery.^61^ It was shown that, in addition to rotation with the local angular velocity of the flow, the particle also has a rotational component that depends on its aspect ratio and orientation as the particle unequally experiences the surrounding velocity field.^61,62^ Furthermore, as shown by Hinch and Leal,^63^ aberrations from the axisymmetric geometry lead to large changes in particle rotation.

Going beyond the ideal 3D geometric shapes mentioned above, in this work, we tackle a biopolymer which is expected to exhibit even more complex behavior (e.g., due to the presence of inertial forces, Brownian motion, and hydrodynamic interactions). To illustrate, in the study of star polymers in solution subjected to shear flow, Ripoll et al.^64^ showed that at very low shear rates, reduced rotational velocity approaches the value of 1/2, while it starts to decrease at higher shear rates. By variation of the number of arms constituting the star, it was observed that the angular velocity becomes (almost) independent of the applied shear rate. On the contrary, Xu and Chen^65^ showed that the latter finding does not hold for the melt of star polymers. Differences in observations of Ripoll et al. and Xu and Chen should be considered through the presence of hydrodynamic and intermolecular interactions. While the former governs the dynamics of dilute solutions (Zimm regime^66^), the latter is more pronounced in polymer melts (Rouse regime^67^), where hydrodynamic interactions are screened.^65^

Therefore, to shed light on the rotational dynamics of the biopolymer, we conduct out-of-equilibrium simulations of ubiquitin using the OBMD method. First, we inspect the effect of the shear flow of various strengths on the apparent angular velocity of the protein. As already pointed out, the apparent angular velocity is not the real one because it includes vibrations with angular momentum. In order to disentangle rotations and vibrations, we employ the Eckart frame formalism. The reference positions of the particles from their center of mass are in the Eckart frame determined from the minimized protein structure. In addition, we also checked whether the angular velocity of the protein combined with EN is altered if the reference configuration is chosen to be an energy-minimized structure or if it is changed after a predefined number of sampled configurations. However, we did not observe significant differences. Furthermore, by altering EN to allow the irreversible breaking of bonds responsible for maintaining the secondary and tertiary structures, we also explore the rotational velocity of the protein that is able to change its conformation from coiled to stretched and vice versa.

**Figure 3:**
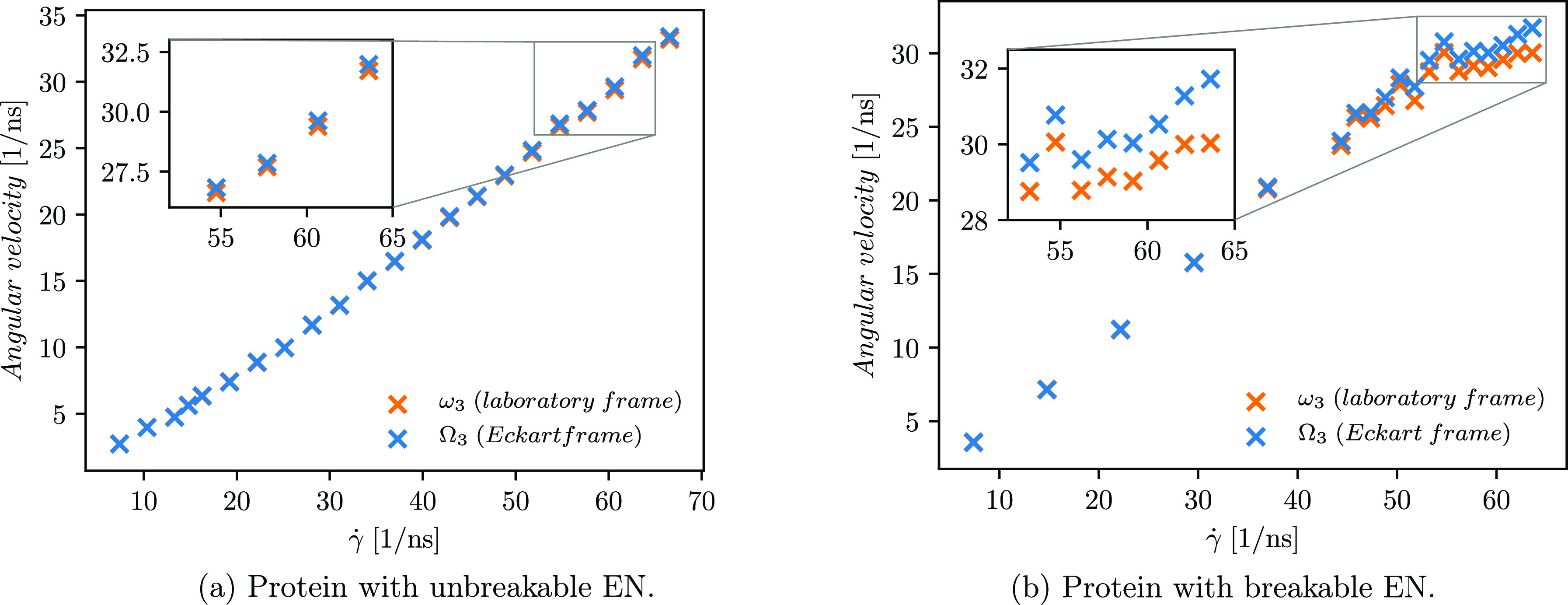
Protein rotation. Orange symbols indicate the results calculated using the standard approach (performed in the laboratory frame), while blue symbols denote the results obtained by employing the Eckart frame formalism. The standard error is less than 3%.

As the protein rotates in the flow–gradient plane, consequently, the only nonzero component of angular velocity (i.e., apparent angular velocity, **ω**, if calculated in the laboratory frame, or **Ω**, if computed using the Eckart frame formalism) is in the vorticity direction (i.e., the *x*_3_-direction). For this reason, Figure 3 shows only the nonzero component of the calculated apparent (i.e., ω_3_) and Eckart (i.e., Ω_3_) angular velocities for the protein with unbreakable (see Figure 3a) and breakable ENs (see Figure 3b). We find that both angular velocities, i.e., ω_3_ and Ω_3_, nonlinearly increase with increasing shear rate. Furthermore, at higher shear rates for the protein with an altered EN, the difference between the angular velocity computed in the laboratory frame and that by employing the Eckart frame formalism is indicated (see the inset plot of Figure 3b). A smaller difference (within the error bar) is observed for the protein combined with EN (see the inset plot of Figure 3a). The observed difference may be understood by the following cogitation. As already emphasized, in the presence of the vibrational angular momentum, the angular velocity determined in the laboratory frame does not properly describe the molecule’s rotational dynamics.^16,18^ For molecules that are more “flexible” or extensible, the vibrational angular momentum contribution is larger.^16^ Therefore, when the protein structure is maintained by the unbreakable EN, it consequently cannot (strongly) deform (or stretch) in the presence of shear flow. Conversely, when breakable EN is used, the protein can now change its conformation with large fluctuations in its extension. The extent to which the protein stretches (in general) depends on the strength of the shear flow. From monitoring the radius of gyration during the production run, it appears that the protein unfolds more frequently and to a greater extent when exposed to higher shear rates (see Figure 6 and red crosses in Figure 7). Accordingly, the contribution of the vibrational angular momentum increases (Figure 11d), and the difference between the apparent and Eckart angular velocities appears (Figure 3b).

**Figure 4:**
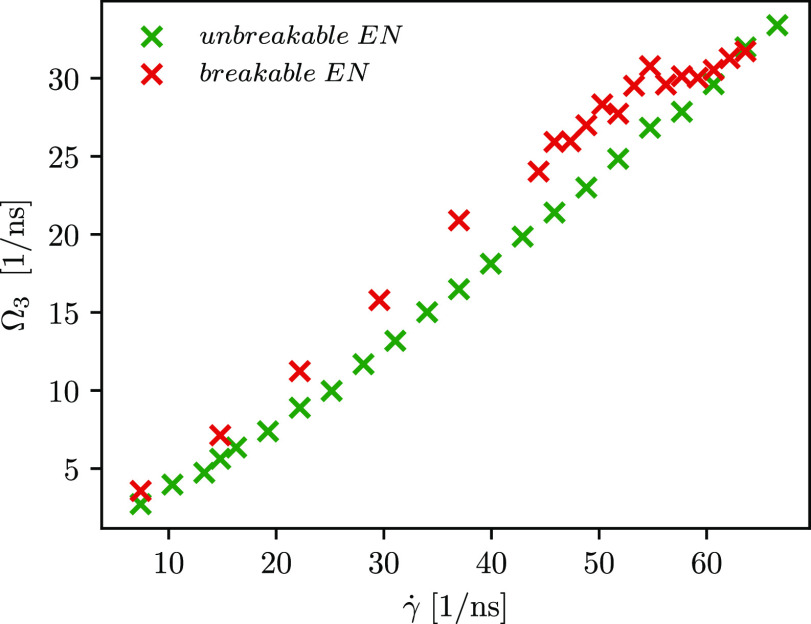
Comparison of the angular velocity computed with Eckart frame. Green and red symbols indicate the results calculated for the protein with unbreakable and breakable ENs, respectively.

**Figure 5:**
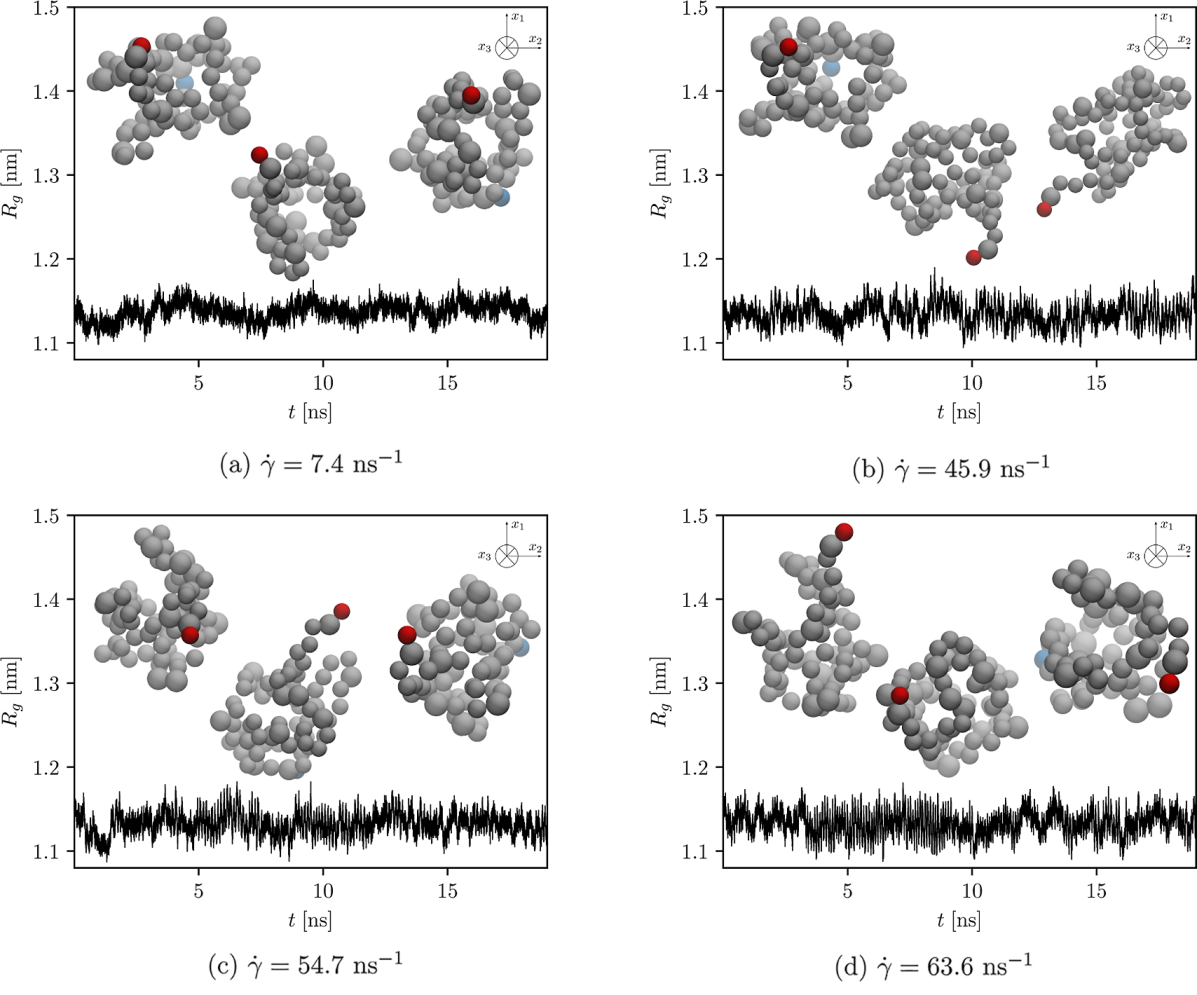
Radius of gyration over time at different shear rates computed for the protein, where its secondary and tertiary structures are preserved by the unbreakable EN. Additionally, representative snapshots are depicted. To better visualize the rotation, only the backbone structure of the protein is shown, with the first particle that makes up the protein colored in blue, the last particle in red, and the rest of the backbone in gray. From the left to right, the first snapshot corresponds to the conformation when the equilibration part of the simulation continues into the production run, i.e., when the velocity profile of the applied shear is expected to be fully developed. In the middle, the protein configuration halfway through the production run is shown, followed by the final configuration at the end of the production run.

**Figure 6:**
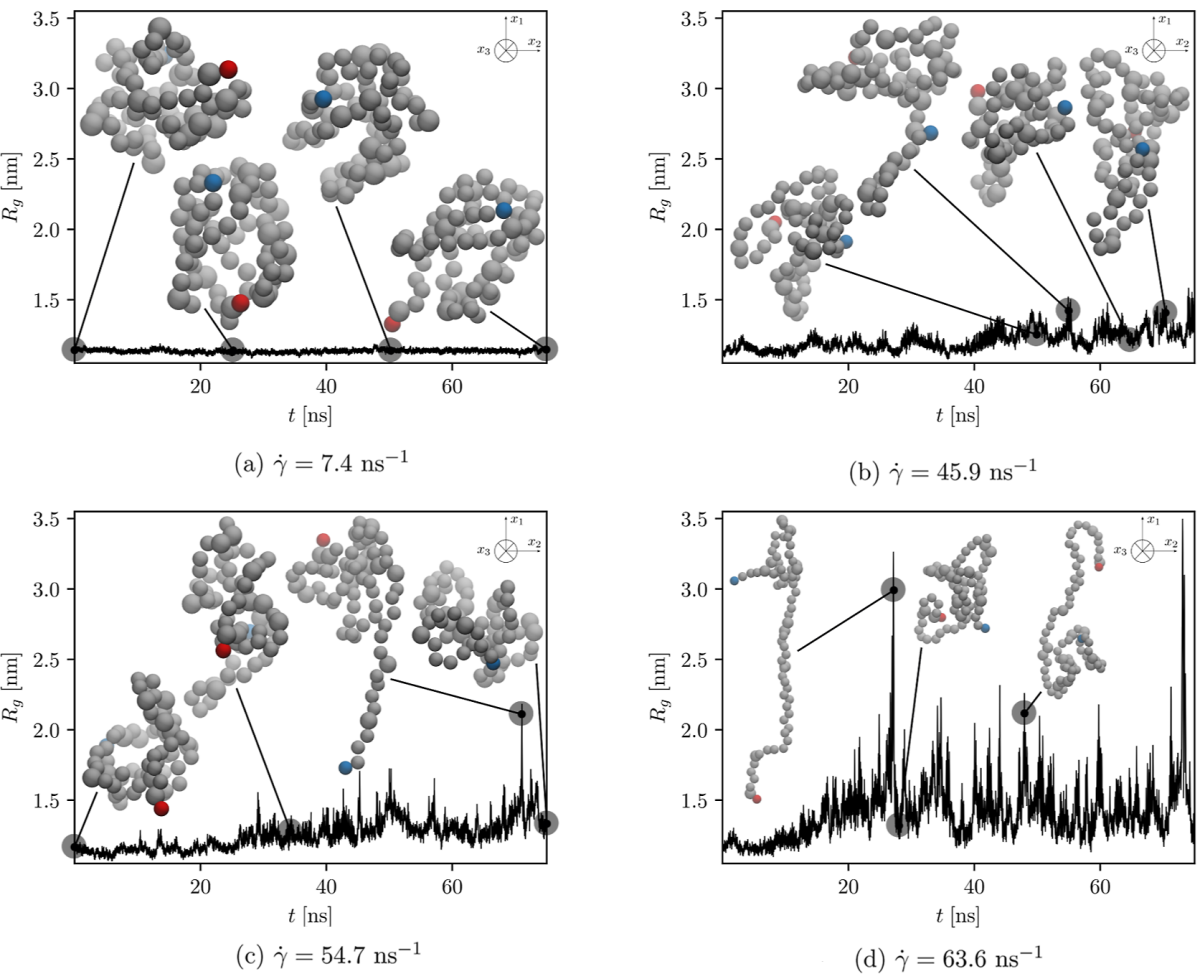
Radius of gyration over time at different shear rates calculated for the protein with the breakable EN. Some selected snapshots, which are considered to best represent the conformational dynamics of the protein, are shown and connected by the black line to the corresponding time of the production run. The coloring of the protein is the same as that in Figure 5.

**Figure 7:**
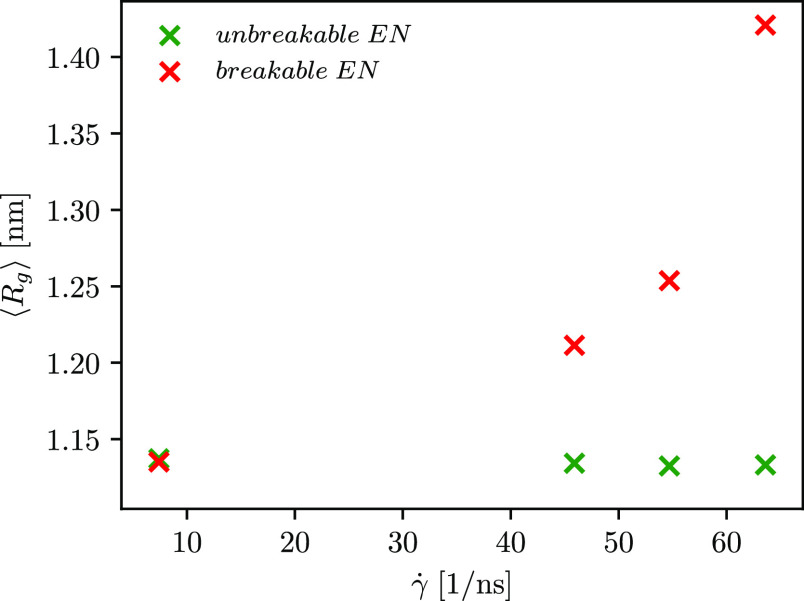
Average radius of gyration at different shear rates calculated for the protein with unbreakable (green symbols) and breakable (red symbols) ENs. Error bars are less than 5%. For the protein with an altered EN, the error bars increase with increasing shear rate.

**Figure 8:**
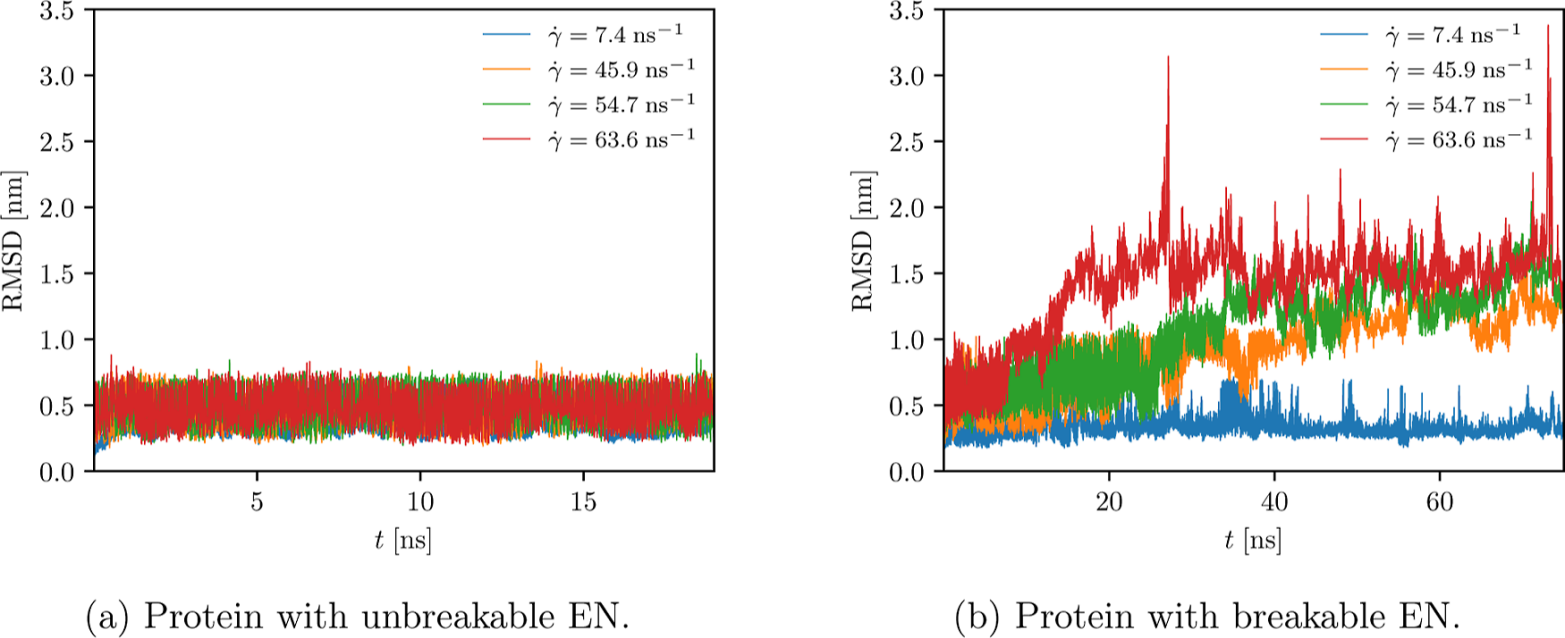
RMSD values of all beads.

**Figure 9:**
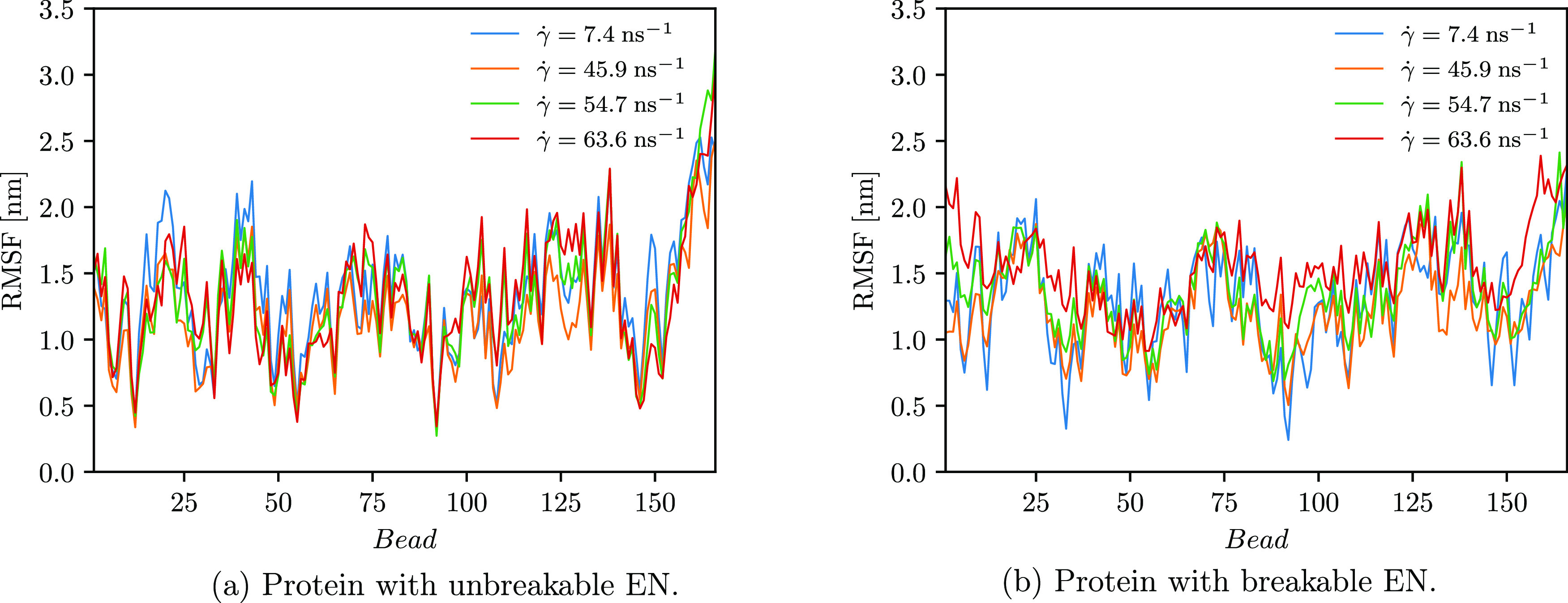
RMSF was computed for each bead.

**Figure 10:**
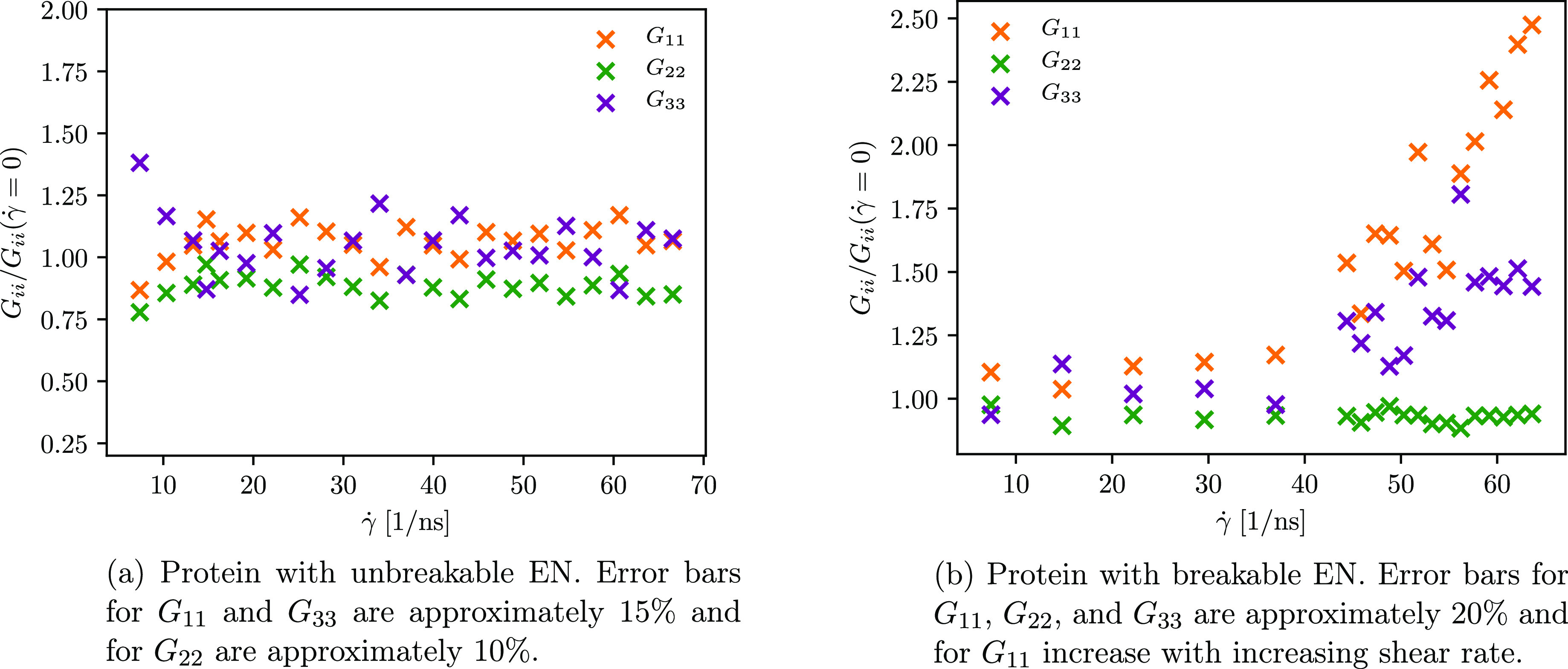
Diagonal components of the averaged gyration tensor (*G*_*ii*_) for the protein with unbreakable and breakable ENs subjected to shear flow of various strengths, normalized by the diagonal components of the averaged gyration tensor for the protein under the zero shear condition (*G*_*ii*_(γ̇ = 0)).

**Figure 11:**
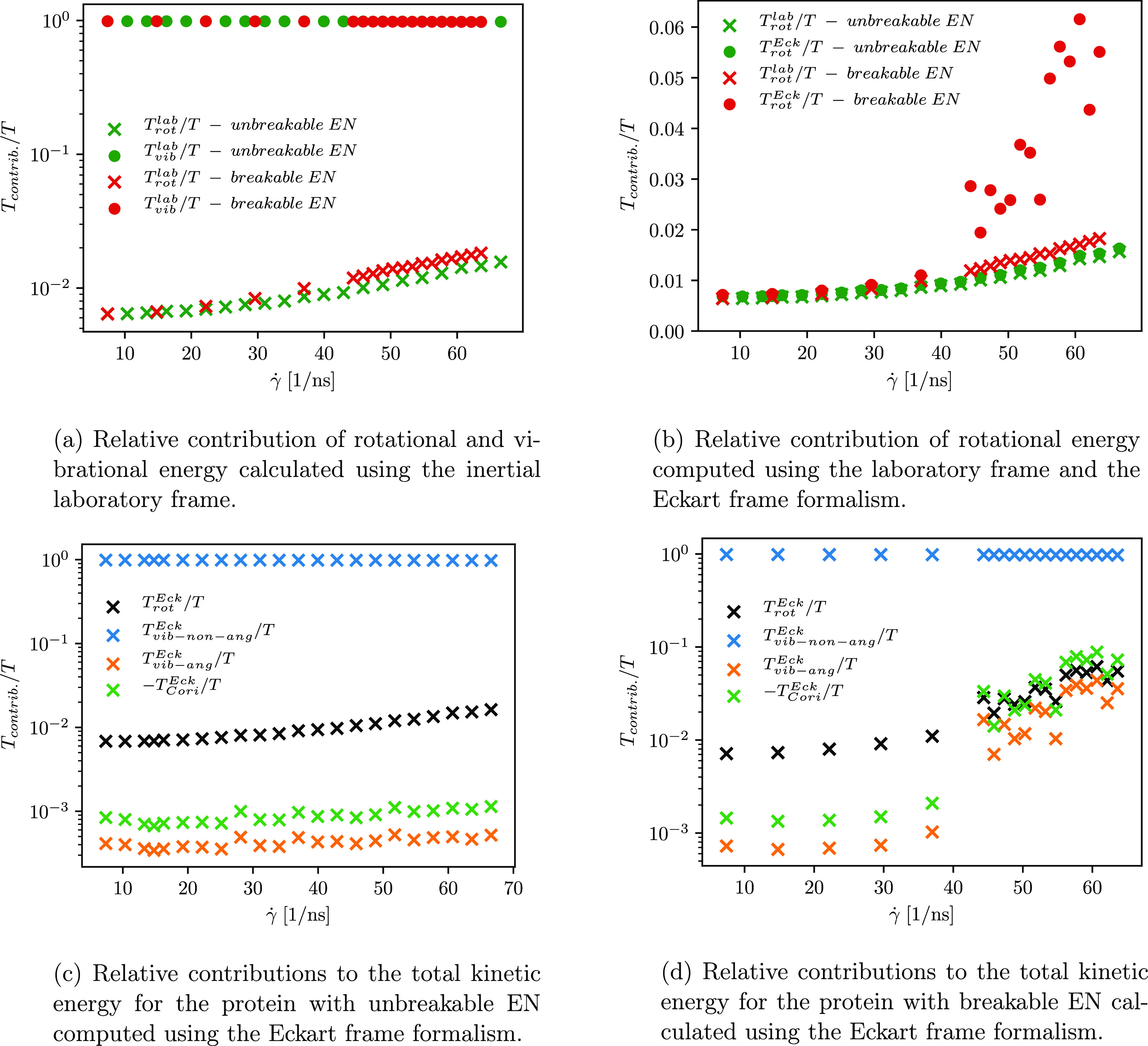
Comparison of the relative contributions to the total kinetic energy of the protein with unbreakable and breakable ENs when a different approach is used to compute the angular velocity.

Due to inquisitiveness about the extent to which the shear flow affects the angular velocity of the protein with unbreakable and breakable ENs, we plot the dependence of the angular velocity on the shear rate for both the cases in Figure 4. As depicted, a higher angular velocity is observed for the protein with a breakable EN, which can be understood by the following consideration of the hydrodynamic drag force. By virtue of the higher velocity gradient at higher shear rates, the extent to which the molecule stretches increases with increasing shear rate, and the net hydrodynamic drag forces acting over the molecule also increase.^12,33,68,69^ The protein with the unbreakable EN is more compact, and its structure is less extended even at higher shear rates. The more extended protein structure is observed for the protein with the breakable EN. Therefore, the parts inside the more compact protein are shielded from the flow, and only a small portion of them experience the full drag force, whereas the protruding parts of the protein with the breakable EN are exposed to larger hydrodynamic drag forces. The latter also leads to the observation of a higher angular velocity for the protein with a breakable EN.

To show vivid conformational dynamics of the protein and confirm our claims, we plot the time evolution of the computed radius of gyration and show some representative simulation snapshots of the protein with unaltered and altered ENs in Figures 5 and 6, respectively. In addition, the average radius of gyration calculated from the selected time evolutions shown in Figures 5 and 6 is depicted in Figure 7.

Our expectation that the protein with an unaltered EN would not unfold is confirmed by the time evolution of the radius of gyration depicted in Figure 5 and by the computed average radius of gyration shown in Figure 7 (see green crosses). It is also substantiated by the computed time evolution of the root-mean-square displacement (RMSD) and root-mean-square fluctuation (RMSF), as shown in Figures 8a and 9a, respectively. As depicted in Figure 5, the radius of gyration only fluctuates around the equilibrium value (of approximately 1.14 nm), regardless of the strength of the applied shear flow (see also green crosses in Figure 7 corresponding to the average radius of gyration). A similar finding applies to the RMSD (Figure 8a), where an increase in RMSD, indicating the unfolded state, is not observed. In addition, no discrepancies in RMSF are observed when the strength of the shear flow is increased (Figure 9a). Therefore, these results imply that the protein remains folded throughout the production run, as expected, since its secondary and tertiary structures are well preserved due to the EN. In contrast, a higher value of the average radius of gyration and many sudden increases in the time evolution of radius of gyration and in RMSD are observed when the protein with the altered EN is subjected to higher shear rates (see Figures 7 (red crosses), 6, and 8b), corresponding to the changes in its conformational state from folded to unfolded (and vice versa). Besides, as depicted in Figure 9b, as the shear rate increases, the groups of beads at the beginning and at the end of the biopolymer (the latter are also further away from the protein’s COM) fluctuate more.

It is well-known that polymers subjected to shear flow are stretched along the flow direction and compressed along the gradient direction.^70^ Besides, the overall conformation of the biopolymer in the flow, gradient, and vorticity direction can be inferred based on the (diagonal) gyration tensor components. Therefore, intrigued by the question of whether a similar behavior can be observed in the case of the protein with EN also, we calculate the gyration tensor using the following equation
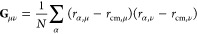
21where μ, ν ∈ (1, 2, 3) stands for the μν component of the gyration tensor. However, in Figure 10a, we show only the diagonal components of the averaged gyration tensor normalized by the diagonal components of the averaged gyration tensor for the protein under zero shear. In general, we can assume that the protein is stretched in the flow direction and shrunk in the gradient direction as the flow-direction component (i.e., 11) on average rises above 1 and the gradient-direction component (i.e., 22) on average decreases below 1. In addition, a minor deformation in the vorticity direction is indicated by the rise of the 33-component of the gyration tensor. However, due to the presence of the unbreakable EN, which “constraints” the protein, these features are not very pronounced. Different conformational dynamics is observed when the protein with the breakable EN is exposed to the shear flow of various strengths. Its conformation is still maintained at the lower shear rate, as depicted in Figures 6a and 8b, but this is not the case when it is exposed to higher shear rates, as shown in Figures 6b–d and 8b. At a low shear rate, the radius of gyration fluctuates around the equilibrium value (as in the case of the protein combined with an unaltered EN), while frequent fluctuations are observed as the shear rate increases. Since a larger radius of gyration is associated with more stretched conformation, we see that the applied shear rate must be high enough to induce the protein to stretch. Based on the diagonal components of the averaged gyration tensor shown in Figure 10b, we assume that the shear rate (for our model) must exceed 40 ns^–1^. The premise that the unfolding takes place at very high shear rates is also in accordance with experimental findings for small globular proteins.^8^ Again, compression and minor deformation of the protein in the gradient and vorticity directions are indicated by the 22- and 33-components of the averaged gyration tensor, respectively. Despite the observations that the exchange of stretched and coiled states occurs more frequently at higher shear rates, where the extent to which the protein unfolds also increases (compare Figure 6a with Figure 6d), we do not observe fully stretched proteins in our simulations (even when applying the highest shear rate, see Figure 6d).

We also calculate the contributions to the total kinetic energy of the rotating molecule when a different approach is employed to compute the angular velocity. Using the laboratory frame, the total kinetic energy is divided into three contributions, i.e., translational, rotational, and vibrational (eq 15). However, it is not possible to differ between pure rotations and vibrations of the molecule because the apparent angular velocity also includes vibrations with angular momentum. For this reason, we aim to use the Eckart frame formalism, which minimizes the coupling between vibrational angular momentum and pure rotation and therefore allows us to discern between vibrations with and without angular momentum (see eq 16). The comparison of the computed contributions (for the protein with unaltered and altered ENs) is shown in Figure 11. Employing the laboratory frame analysis in the angular velocity calculation, we observe that the contribution of the vibrational energy is comparable for both cases when the EN of the protein remains unaltered or it is altered (see green and red dots in Figure 11a). On the contrary, a smaller difference in the rotational energy contribution is observed for higher shear rates (see green and red crosses in Figure 11a).

As previously discussed, when the protein with the breakable EN is subjected to stronger shear flow, its conformation changes with large fluctuations in its extension. This results in an increase in the vibrational contribution with angular momentum, which is part of the rotational energy (see eq 20), and is therefore reflected in the observation of a slightly higher rotational energy contribution when the protein with altered EN is subjected to the stronger shear rate. An increase in the vibrational angular momentum of the protein with an altered EN can be seen in Figure 11d. Furthermore, using the Eckart frame in the angular velocity calculation, a larger rotational contribution is observed compared to that determined from the apparent angular velocity computation (Figure 11b). As shown in Figure 3b, the Eckart angular velocity is also larger compared to the apparent angular velocity, which is manifested in the larger rotational energy (see the second term on the rhs of eq 16). As evident from Figure 11c,d, the vibrations without angular momentum have the largest contribution to the total kinetic energy of the rotating protein molecule with unbreakable and breakable ENs. An increase in the vibrational velocity contribution of constituting particles, i.e., **u**_α_, that is part of the contribution with angular momentum, also gives rise to the Coriolis contribution. Moreover, when the difference between the Eckart and the apparent angular velocity increases, the Coriolis contribution also becomes more negative (see Figure 11d, where the absolute value of the Coriolis term is depicted).

The implementation of an additional spring is essential in keeping the protein close to the center of the simulation box and preventing it from diffusing through the open edges of the simulation box, thus preserving its geometry and preventing it from being erased (if it crosses the open ends of the simulation domain). Additionally, this also results in a restriction of the translational movement of the protein. We assume that without this constraint, the trajectory of the protein would deviate in one of the two directions due to the flow-induced hydrodynamic lift force (acting on the particle in the direction that is perpendicular to the flow). We expect this to eventually push the protein into higher velocity streams, i.e., in the proximity of the buffer region. This effect is compensated for in OBMD simulations with the implemented spring. The effect of the spring on the motion of the biomolecule could be estimated from the force obtained from the known (applied) spring constant and from monitoring the magnitude of the displacement of the protein’s COM from the center of the simulation box. Furthermore, as the Martini 3 protein model combined with EN does not permit the investigation of unfolding and refolding events, we modify EN by specifying the cutoff values for the distances at which the bonds between CG bead pairs of EN are irreversibly broken. The cutoff value used in this study is the one at which the protein’s structure is maintained during the equilibrium simulation (based on monitoring of the radius of gyration). Larger cutoff values would imply the breaking of EN bonds at higher shear rates, while lower values would not maintain a stable conformation.

## 5 Conclusions

The main goal of this study was to inspect and understand the effect of induced mechanical stress introduced through the shear flow on the rotational and conformational dynamics of proteins. To this end, the protein ubiquitin (described by the CG Martini 3 model with unbreakable and breakable ENs) was subjected to the shear flow of various strengths. Its rotational dynamics was explored using the standard laboratory frame description and Eckart frame formalism. The angular velocity extracted in the laboratory frame mixes pure rotation and vibration with angular momentum and thus has no clear dynamic interpretation. To correctly describe the rotational and vibrational motions of the biomolecule, we employed the Eckart frame formalism, in which the coupling between pure rotation and vibrational angular momentum was minimized. As shown, at higher shear rates, the contribution of vibrations with angular momentum is greater, which is also reflected in the observed difference between the apparent and Eckart angular velocities. Besides, in the protein with a breakable EN (that is also more “flexible”), the protruding parts experience larger hydrodynamic drag forces, whereas in a more compact protein with the unbreakable EN, this applies to only a small portion of the segments.

We focused on the conformational dynamics of the protein with a breakable EN when exposed to shear flow of greater strengths. In general, we observed that the protein is stretched in the flow direction and compressed in the gradient one. Minor deformation was also observed in the vorticity direction. Additionally, in this work, we did not observe a fully stretched protein (regardless of the strength of the induced shear).

Due to the essential role of proteins both in biological processes and in bioprocessing, it is necessary to properly perform an inspection of their behavior in rotational and conformational space. During certain steps in the production of therapeutic proteins, they are subjected to shear stress, which can lead to undesirable consequences, such as reduced therapeutic efficacy, activity, and aggregation. However, many contrasting results concerning the effects of shear are found in the literature. As it appears, addressing this question is not unequivocal and is still imperative. In the present study, by determining the pure angular velocity of the protein, we provided another perspective to understand the susceptibility of the protein to the induced shear stress, leading to a better interpretation of the dynamics of the rotating and vibrating biologically relevant molecule. Future work would benefit from the use of the all-atom molecular models to give even better insight into the conformational and rotational dynamics addressed in this work and from the search for new approaches to circumvent the limitations (regarding the translational freedom of the biomolecule) presented in this study.
